# Supplementary material for: “Separated during the first hours”—Postnatal care for women and newborns during the COVID-19 pandemic: A mixed-methods cross-sectional study from a global online survey of maternal and newborn healthcare providers
Source: PLOS Glob Public Health. 2022 Apr 28;2(4):e0000214. doi: 10.1371/journal.pgph.0000214 (PMC10022345; doi:10.1371/journal.pgph.0000214)
Supplement: S1 Table — (DOCX) [file pgph.0000214.s001.docx]

**S1 Table - Survey questions included in the analysis**

**List of background questions**

| *Q#* | *Question* | *Response* |
| --- | --- | --- |
| 1 | In which **country** are you based (providing healthcare) at the moment? | [drop down menu of countries] |
| 2 | What is your main **job**?  (choose one) | Midwife  Nurse-midwife  Nurse  Obstetrician/gynaecologist  Neonatologist  Paediatrician  Medical Doctor  Other (Please specify other job) |
| 3 | What is your **position**?  (choose one) | Head of facility (director, administrator)  Head of department or ward  Head of team  Team member  Locum or interim member  Independent or self-practicing  Other (Please specify other position) |
| 4 | What **type of maternal and/or neonatal health care** do you currently provide as an individual?  (select all that apply) | Outpatient antenatal care  Outpatient (home-based) childbirth care  Outpatient postnatal care  Outpatient breastfeeding support  Inpatient antenatal care  Inpatient childbirth care  Inpatient postnatal care (mother and/or babies)  Surgical care  Neonatal care for small and sick newborns  Home visits  Community outreach, home visits, health education outside facility  Family planning provision or counselling  Abortion care  Post-abortion care  Other (Please specify other care type) |

**List of questions about the setting and place of work**

| Q# | Question | Response |
| --- | --- | --- |
| 1 | In which **level** of health care institution do **you primarily work**?  Select the one where you spend most of your time.  (if none of the response options fit well, please use the “Other” option and write what your facility type is called in your country) | Referral hospital  District/regional hospital  Health centre  Polyclinic or Clinic  Birth centre  Health post/unit  Dispensary  Home-based care  Independent/self-practicing  Other (Please specify) |
| 2 | What organisation **type** is your **primary** institution or employer?  (if none of the response options fit well, please use the “Other” option and write what your organisation type is called in your country) | Public (national)  Public (university or teaching)  Public (district level or lower)  Social security  Health insurance  Private university  Private not-for-profit  Private for profit  Non-governmental  Faith-based or mission  Self-practicing  Other (Please specify) |
| 3 | In what type of **geographic area** do you provide care at the moment? | Large city (>1 mil inhabitants)  Small city (100,000 to 1 mil inhabitants)  Town (<100,000 inhabitants)  Village or rural area  Refugee or displaced persons camp  Other (Please specify) |

You have now completed the main part of the questionnaire. If you have more time, we would like to ask 11 additional questions about how the provision of care in your facility and community has been affected by COVID-19. Please select “I agree” if you would like to continue to this additional last section of this survey.

__ I agree to continue to the additional module

__ I would like to end the survey

**Additional module questions: Effect of COVID-19 on the provision of maternal and newborn care**

| Q# | Question | Response |
| --- | --- | --- |
| First, we would like to ask how the **provision** of the various types of maternal and newborn care has been affected in the past month. | | |
| 4 | In the past month, how was **inpatient postnatal care for women and newborns** affected?  Please select all that apply | - Suspended provision completely for some or all of the time - Shorter operating hours or reduced number of days care is available - Reduced space on the postnatal ward due to creation of COVID-19 isolation rooms/ spaces - Dedicated neonatal cots/equipment for newborns born to COVID-19 mothers - Reduced number of beds due to social distancing measures - Reduced number of allowed visitors - Visitors banned - Shortened visiting hours - Parents not allowed to visit newborn in newborn intensive care units (NICU) - Other changes (please describe other changes) |
| 5 | In the past month, how was **outpatient postnatal** care affected?  Please select all that apply | - Suspended provision completely for some or all of the time - Shorter operating hours or reduced number of days care is available - Prioritising highest need patients only - Patients appointments scheduled further apart - Unable to see all patients in person - Use of telemedicine to provide care - Reduced number of women/newborns accessing care - Home-based visits reduced or stopped - Other changes (please describe other changes) |
|  | Next, we would like to ask about changes you noticed in the **content of care**. | |
| 9 | In the past month, was the content of **inpatient** **postnatal** care to women and newborns provided by you or in your facility affected in terms of:  (select all that apply) | - Shorter length of stay in facility / earlier discharge - Less frequent routine postnatal monitoring in the facility - Reduced / suspended newborn vaccination or screening - Limited skin-to skin contact between newborn and mother - Delayed initiation of breastfeeding - COVID-19 suspected or positive mothers: separating mother and baby - COVID-19 suspected or positive mothers: breastfeeding not allowed or discouraged - Other changes (please describe other changes) |
| 10 | In the past month, was the content of **outpatient postnatal** care to women and newborns provided by you or in your facility affected in terms of:  (select all that apply) | - Reduced/ suspended breastfeeding support - Reduced/suspended postpartum family planning counselling / provision - Reduced/ suspended newborn vaccination - Reduced/ suspended newborn weight monitoring - Reduced/ suspended mental health monitoring and support to women - Reduced/ suspended social care support or referral - Other changes (please describe other changes) |
