# Supplementary material for: “Separated during the first hours”—Postnatal care for women and newborns during the COVID-19 pandemic: A mixed-methods cross-sectional study from a global online survey of maternal and newborn healthcare providers
Source: PLOS Glob Public Health. 2022 Apr 28;2(4):e0000214. doi: 10.1371/journal.pgph.0000214 (PMC10022345; doi:10.1371/journal.pgph.0000214)
Supplement: S2 Table — (DOCX) [file pgph.0000214.s002.docx]

**S2 Table – Country distribution of maternal and newborn healthcare providers (n=424)**

| **High income countries** | **193 (45.5)** |  | **Lower middle income countries** | **91 (21.5)** |
| --- | --- | --- | --- | --- |
| Australia | 1 (0.5) |  | Bangladesh | 2 (2.4) |
| Austria | 1 (0.5) |  | Bolivia | 5 (6.0) |
| Barbados | 1 (0.5) |  | Cameroon | 18 (21.4) |
| Belgium | 12 (6.2) |  | Côte d’Ivoire | 1 (1.2) |
| Canada | 8 (4.1) |  | India | 12 (14.3) |
| Chile | 1 (0.5) |  | Kenya | 6 (7.1) |
| Denmark | 1 (0.5) |  | Morocco | 12 (14.3) |
| Estonia | 1 (0.5) |  | Myanmar (Burma) | 1 (1.2) |
| France | 3 (1.6) |  | Nicaragua | 2 (2.4) |
| Germany | 22 (11.3) |  | Nigeria | 18 (21.4) |
| Iceland | 1 (0.5) |  | Philippines | 1 (1.2) |
| Ireland | 1 (0.5) |  | Tanzania | 7 (12.3) |
| Italy | 36 (18.6) |  | Uzbekistan | 1 (1.2) |
| Japan | 31 (16.0) |  | Vietnam | 1 (1.2) |
| Netherlands | 1 (0.5) |  | Zambia | 4 (4.8) |
| New Zealand | 2 (1.0) |  |  |  |
| Norway | 9 (4.6) |  | **Low income countries** | **50 (11.8)** |
| Portugal | 4 (2.0) |  | Afghanistan | 1 (1.8) |
| Saudi Arabia | 4 (2.0) |  | Benin | 1 (1.8) |
| Slovakia | 2 (1.0) |  | Democratic Republic of the Congo | 31 (54.4) |
| Spain | 6 (3.1) |  | Ethiopia | 1 (1.8) |
| Sweden | 1 (0.5) |  | Guinea | 4 (7.0) |
| Switzerland | 9 (4.6) |  | Malawi | 1 (1.8) |
| Trinidad and Tobago | 2 (1.0) |  | Mozambique | 1 (1.8) |
| United Kingdom | 8 (4.1) |  | Nepal | 1 (1.8) |
| United States | 10 (5.1) |  | Rwanda | 1 (1.8) |
| Uruguay | 15 (7.7) |  | Syria | 1 (1.8) |
|  |  |  | Uganda | 7 (12.3) |
| **Upper middle income countries** | **90 (21.2)** |  |  |  |
| Argentina | 9 (10.1) |  |  |  |
| Brazil | 6 (6.7) |  |  |  |
| Costa Rica | 1 (1.1) |  |  |  |
| Dominican Republic | 1 (1.1) |  |  |  |
| Kazakhstan | 68 (76.4) |  |  |  |
| Panama | 1 (0.5) |  |  |  |
| Romania | 1 (1.1) |  |  |  |
| South Africa | 3 (3.4) |  |  |  |
