# Supplementary material for: “Separated during the first hours”—Postnatal care for women and newborns during the COVID-19 pandemic: A mixed-methods cross-sectional study from a global online survey of maternal and newborn healthcare providers
Source: PLOS Glob Public Health. 2022 Apr 28;2(4):e0000214. doi: 10.1371/journal.pgph.0000214 (PMC10022345; doi:10.1371/journal.pgph.0000214)
Supplement: S3 Table — (DOCX) [file pgph.0000214.s003.docx]

**S3 Table –** **Analysis disaggregated by type of care provided by respondents (PNC vs no PNC)**

|  | **PNC**  **n (%)** | **No PNC**  **n (%)** | **Total**  **n (%)** |
| --- | --- | --- | --- |
| **Adaptations in the process of PNC to reduce SARS-CoV-2 risk** |  |  |  |
| Reduced number of allowed visitors in health facilities | 160 (57.1) | 55 (55) | 215 (56.6) |
| Visitors banned from health facilities | 126 (45) | 38 (38) | 164 (43.2) |
| Shortened visiting hours in health facilities | 116 (41.4) | 30 (30) | 146 (38.4) |
| Appointments scheduled further apart for consecutive outpatients | 114 (44) | 30 (33.3) | 144 (41.3) |
| Telemedicine to provide outpatient postnatal care | 78 (30.1) | 16 (17.8) | 94 (26.9) |
| Reduced number of beds due to social distancing measures | 59 (21.1) | 22 (22) | 81 (21.3) |
| Reduced space on postnatal ward due to COVID-19 isolation rooms | 53 (18.9) | 24 (24) | 77 (20.3) |
| Parents not allowed to visit newborn in NICU | 34 (12.1) | 13 (13) | 47 (12.4) |
| **Changes in PNC provision and service availability** |  |  |  |
| Prioritising highest need outpatients for face-to-face postnatal visits | 89 (34.4) | 29 (32.2) | 118 (33.8) |
| Reduced number of women/newborns accessing outpatient postnatal care | 75 (29) | 22 (24.4) | 97 (27.8) |
| Unable to provide postnatal care face to face to all outpatients | 52 (20.1) | 18 (20) | 70 (20.1) |
| Home-based visits reduced or stopped | 50 (19.3) | 13 (14.4) | 63 (18.1) |
| Shorter operating hours or less days outpatient postnatal care is available | 57 (22) | 18 (20) | 75 (21.5) |
| Suspended provision of outpatient postnatal care | 40 (15.4) | 18 (20) | 58 (16.6) |
| Shorter operating hours or less days inpatient postnatal care is available | 44 (15.7) | 12 (12) | 56 (14.7) |
| Suspended provision of inpatient postnatal care | 24 (8.6) | 14 (14) | 38 (10) |
| **Changes in PNC content** |  |  |  |
| Shorter length of stay in facility/earlier discharge | 129 (59.2) | 42 (54.5) | 171 (58) |
| Less frequent routine postnatal monitoring in the facility | 61 (28) | 15 (19.5) | 76 (25.8) |
| Reduced duration or content of home based postnatal visits | 73 (38.4) | 16 (26.7) | 89 (35.6) |
| Reduced/suspended postpartum family planning counselling/provision | 51 (26.8) | 23 (38.3) | 74 (29.6) |
| Reduced/suspended breastfeeding support to women | 53 (27.9) | 15 (25) | 68 (27.2) |
| Reduced/suspended social care support or referral | 52 (27.4) | 10 (16.7) | 62 (24.8) |
| Reduced/suspended mental health monitoring and support to women | 45 (23.7) | 13 (21.7) | 58 (23.2) |
| Reduced/suspended newborn weight monitoring | 41 (21.6) | 13 (21.7) | 54 (21.6) |
| Reduced/suspended newborn vaccination (outpatient) | 34 (17.9) | 15 (25) | 49 (19.6) |
| Limited skin-to-skin contact between mother and newborn | 28 (12.8) | 11 (14.3) | 39 (13.2) |
| Reduced/suspended newborn vaccination or screening (inpatient) | 25 (11.5) | 6 (7.8) | 31 (10.5) |
| Delayed initiation of breastfeeding | 15 (6.9) | 5 (6.5) | 20 (6.8) |
| **PNC for newborns and mothers suspected/confirmed with COVID-19** |  |  |  |
| Separating mother and baby | 60 (27.5) | 19 (24.7) | 79 (26.8) |
| Breastfeeding not allowed or discouraged | 26 (11.9) | 11 (14.3) | 37 (12.5) |
| Special cots/equipment for newborns of mothers with COVID-19 | 49 (17.5) | 13 (13) | 62 (16.3) |
| **Total** | **311 (74)** | **111 (26)** | **422* (100)** |

*The sample size is smaller than that of the full analysis due to missing answers to the question on the type of care provided.

PNC = Postnatal care
