# Supplementary material for: “Separated during the first hours”—Postnatal care for women and newborns during the COVID-19 pandemic: A mixed-methods cross-sectional study from a global online survey of maternal and newborn healthcare providers
Source: PLOS Glob Public Health. 2022 Apr 28;2(4):e0000214. doi: 10.1371/journal.pgph.0000214 (PMC10022345; doi:10.1371/journal.pgph.0000214)
Supplement: Attachment — Submitted filename: Response to reviewers_23112021.docx [file pgph.0000214.s004.docx]

**Response to Reviewer #1:**

**Thank you for the opportunity of reviewing your manuscript. Even though the subject is of interest to me, I did not quite understand what you tried to show in this article. Indeed initial PNC coverage i.e. before the pandemic was not studied it is therefore complicated to conclude that COVID 19 has modified this follow-up. Moreover, the consequences of the potential modification in PNC have not been studied.**

- Thanks for your feedback. This study does not attempt to systematically compare PNC coverage before and during the COVID-19 pandemic, and we do not conclude that coverage was modified during COVID-19. Our study documents alterations made to provision of PNC (based on the answers to the questionnaire shown in Supplementary file 1 which specifically asked healthcare providers to report changes in PNC provision and *perceived changes in use*). We found that several of the reported adaptations reflect sub-optimal practices. Conclusions on modifications to PNC are based on the respondents’ perspectives of perceived modifications, and the aim is not to compare with pre-existing practice.
- Key of modifications to PNC provision were discussed in detail in the manuscript as follows:
  - Decline in length-of-stay after birth (line 402 - 415)
  - Telemedicine use (line 471 - 477)
  - Reduced support from visitors and companions and lack of mental health support (line 427-442)
- We have addressed your comments below and believe the totality of the changes made in response to your helpful comments clarify the objective and scope of our inquiry.

**Lines 111-112 "We present findings from a global online survey conducted after the first wave of the COVID-19 pandemic and the concomitant emergency response." This sentence is unclear, please clarify what the authors mean by concomitant emergency response.**

- Thank you for this comment, we have changed the phrase “*concomitant emergency response*” to “*mitigation measures implemented to control the spread of COVID-19*”, which include strict lockdowns, travel bans and interruption to public transport systems, curfews and closure of health facilities in certain countries.
- We also clarified the first section of this sentence as such: “*This study’s objective was to fill this knowledge gap using data from a mixed methods global online survey of maternal and newborn healthcare providers conducted after the early phase of the pandemic (between July and December 2020).”* (line 118 - 124)

**The introduction is too long, gives both a lot of general data with no link to the study (lines 56-73) and very (too?) specific data (lines 85-95). At the end of the introduction the purpose of the study and what it will add to literature is imprecise.**

- General data: we believe that having a paragraph that introduces the details of the components of PNC and existing recommendations regarding PNC service organisation is essential for building a case for readers, who may not be familiar with the specificities of PNC. We also believe that this paragraph is linked to the study as it makes the full-text coherent for the reader since the results include a lot of details that describe specific changes in the organisation of care. Therefore, this paragraph improves the reader’s ability to interpret the findings and implications of the identified practices in PNC during the COVID-19 pandemic. We therefore prefer to retain this general, yet crucial, paragraph in the introduction section. However, we agree that it is long and have shortened it as suggested by the reviewer.
- Indeed the introduction was structured in a way to move from the general and the broad definition and description of PNC to the specific theme on PNC during COVID-19. Hence the paragraph in lines 91-103 is a summary of the existing literature on PNC during the pandemic and is directly linked to the study and frames the paper in light of available knowledge and evidence. We have updated this paragraph since the time that we wrote the manuscript to include an additional paper that was published during our manuscript’s review process. We then specifically move to describing the current evidence on PNC provision to women with suspected/confirmed COVID-19 in the following paragraph (line 104 - 114).
- After the literature review, we highlight knowledge gaps: data on PNC during COVID-19 are strictly quantitative in nature and were collected in the early phase of the pandemic (between March and June 2020). We added a sentence to specify the knowledge gaps that this paper fills (line 115 - 121) by using qualitative and quantitative data from a period beyond the early phase of the pandemic.

**The objective of the study is very vague, the aim was just to describe how PNC was organized during the COVID 19 pandemics? If so, why compare countries according to their incomes?**

- We thank the reviewer for this comment and have revised the objectives listed at the end of the introduction (line 115 - 128). The available literature on the organisation of PNC before the COVID-19 pandemic, as well as responses to COVID-19 (mitigation measures in general and adaptations to provision of maternity care in particular) allowed us to hypothesise that there would be substantive and informative variation across countries in the manner in which provision of PNC was affected. Additionally, stratification across income groups is standard and recommended practice when presenting global health research. This stratification allows us to understand the findings contextually, taking into consideration pre-existing resources and capacities.

**Method section: the lack of details on how the analyses were conducted did not help me understand the objective of this study.**

- We elaborated with an additional sentence about how the qualitative analysis was conducted in this study (line 186 - 187). And we hope that the above-mentioned changes to the objectives are sufficient to clarify the study’s objectives.

**I am quite puzzled after reading this article, the data is interesting but the way this article is written does not highlight the usefulness of these data.**

- One of the strengths of our study is triangulation of qualitative and quantitative data types as much and using quotes as examples that support the quantitative data in many occurrences throughout the manuscript. Our edits have hopefully helped to highlight the usefulness of these data despite the study limitations highlighted in the discussion section (line 494 - 517). To strengthen analysis and interpretation, and to contextualise the findings, the results were disaggregated by country-income group. This study fills a significant data gap that exists globally during the COVID-19 pandemic, specifically for the postnatal period; a time when the highest mortality and morbidity occurs for mother and newborn. As such the data is critical for direct policy action, in building back health systems but equally in future pandemic preparedness plans.

**Response to Reviewer #2**:

**This paper describes postnatal care service availability, content and quality during the COVID-19 pandemic, using quantitative and qualitative data from healthcare providers in 61 countries. Results show significant disruptions to the content and quality of postnatal care, with notable differences by country income groups. These are important findings and the manuscript is well written. The analysis is descriptive, which is adequate. There are only few inaccuracies to consider:**

- **Page 6, line 159: 443 out of 1405 is not 30% (but 31.5%)**
- **Page 6, line 160: 424 out of 443 is not 99% (but 95.7%)**
- **Page 10, line 205: Please provide absolute numbers (n) for these percentages.**
- **Page 11, Box 1: 91 out of 346 is not 35% (but 26.3%)**
- **Page 11, line 230: Text says 36%, figure says 37%. Please correct.**
- **Page 12, lines 243 + 250: Please provide absolute numbers (n) for these percentages.**
- **Page 13, lines 282 + 283 + 294: Please provide absolute numbers (n) for these percentages.**
- **Page 13, line 290: Text says 44%, figure says 45%. Please correct.**
- **Page 14, line 316: "the facility were they worked" should be "the facility where they worked". Please correct.**
- **Figures 1 to 4: Please provide absolute numbers (n) for all percentages in these figures.**

**Please revise and check all remaining numbers and percentages twice!**

- We thank Reviewer #2 for this precise and highly detailed review of the manuscript which helped us improve the quality of our reporting. We appreciate the clarifications and we have edited the manuscript and made corrections to the errors that were identified.

We would like to clarify that within the survey data there was a varying number of missing values for each question/variable. This means that while the total number of respondents (maternity healthcare providers) included in the analysis is 424, the number of respondents who answered each question varied from (is less than) the total. We have now clarified this in the manuscript (line 181 - 183), and added the sample size and absolute numbers when reporting all frequencies and percentages in the figures, tables and in the main-text of the manuscript.

**Response to Reviewer #5**

**This study used a questionnaire with a large number of healthcare providers (424) across 61 countries to determine if there were changes in routine post-natal care due to Covid-19. The abstract is clear and concise. They claim and provide evidence for a reduction in postnatal care services and provide a good set of evidence that these reductions could increase the risks of poor health outcomes. The data supports the claims generally apart from some minor issues mentioned below. The questions used in the survey are provided which is a nice change and enough information is given about the methods used for reproducibility. Original data was not provided due to some small cell counts. I am not sure that the anonymity of respondents would be truly affected unless there was less than 5 workers at a facility. The authors might consider releasing data from larger institutions although I am not sure if that would ultimately provide much as the results section is comprehensive. How this study fits into the existing literature is dealt with in depth in both the Introduction and Discussion. The manuscript is well organised and understandable. The Tables and Figures are useful.**

- We thank Reviewer #5 for their thoughtful review and feedback. Regarding the possibility of sharing individual-level data from larger facilities, we agree that a sub-set of the data would not be of value as it is not sufficient for reproducing the study, while at the same time it would compromise respondents’ anonymity. Following is a hypothetical example of how the raw data can make a respondent identifiable: An obstetrician/gynaecologist who is the head of a primary healthcare centre in a rural area of Country X, wrote in one of the full-text responses that their facility closed for 2 weeks because of a COVID-19 outbreak among the staff. In such a case it will be easy to trace this respondent for readers from their facility, region or country. Protecting our respondents’ anonymity and privacy is of utmost priority and we appreciate your understanding of these ethical standards and values.
- We appreciate the note from the reviewer that they like the survey instrument being made public in Supplementary file 1. Our team has shared survey instruments from the study on our website, and we will consider releasing the dataset for the use of other researchers in the future, in consultation with our ethics committee on any blinding or anonymisation required.

**A few specific thoughts which might help explain a few details for the reader are listed below.**

**Abstract: Mentions “Early initiation of breastfeeding was delayed due to waiting for maternal SARS-CoV-2 test results.” Figure 3 suggests that this affected <10% of countries. However, in the text at line 280 it says “On the other hand, reduction in the provision of breastfeeding support was reported by 281 40% of respondents in high-income countries, compared to 7% in low-income countries”. For me this second set of facts would be more relevant for inclusion in the abstract as it was reported by a larger proportion of respondents.**

- We agree and included the reviewer’s suggestion in the abstract (line 38 - 40).

**Line 85: Looking at Table 9 in the reference provided [22] it appears to show that 33% of countries reported disruption in post-natal care with 2% more than 50% disrupted. I would encourage the authors to check the accuracy of the sentence provided. This fact is again repeated at line 339 in the discussion which may also need to be altered.**

- We thank the reviewer for their thorough review and we have clarified and edited the sentences referring to the second WHO pulse survey in both the introduction (line 91 - 93) and the discussion (line 365 - 366).

**Line 160: You state that you included data from 424 of 443 providers that responded to the optional questionnaire component. To me that comes out to be about 95.7% rather than the 99% reported. Is the n or the proportion reported incorrectly?**

- Thank you for this remark which was also noted by Reviewer #2. We reviewed and corrected the reported percentage to 95.7% (line 174).

**Line 175: How were final decisions made about what codes to use? Consensus? Majority?**

- We clarified in the methods’ section that “*A final list of codes was reached and a coding framework describing the changes and adaptations made to PNC was developed by consensus following a discussion among the study team.*” (line 185 - 188)

**Line 188: If a quarter of respondents didn’t provide any PNC how were they qualified to comment on the PNC provided? Was it because they were managers or heads of team or something similar? It may increase confidence in the readers if you could make this clearer. Have now seen that this is addressed at line 470 that they may have had contact with other staff who could have shared information with them. I feel that hearsay evidence would be less reliable than expected from a questionnaire of this nature. Would it be possible to provide any further analysis/evidence of the similarity of data provided by the cohort which wasn’t involved with PNC directly compared with those that were to ensure consistency?**

- We thank the reviewer for this comment and have taken the suggestion into consideration, which made our reporting more robust. We added in Supplementary file 3 a table of the findings disaggregated by whether respondents provided postnatal care services or not. The results show that for the majority of the outcomes, the results are comparable between the two groups. This suggests that maternity healthcare workers who are not directly involved in postnatal care report similar answers as those healthcare providers who are involved in postnatal care. This explanation was added to the manuscript (line 503 - 508).

**Line 330: “Findings show significant disruptions to the content and quality of PNC, with notable differences by country income groups.” This is similar to what is mentioned in the abstract. However, at line 242 states “Changes to PNC availability and use were not commonly reported and mainly included the prioritisation of providing care to patients with the highest needs (33%)” .Perhaps you could make it clearer in the abstract that content and quality was affected but not nearly as much as availability and use.**

- Thank you. We edited the abstract to reflect the above by adding this sentence: “*Beyond the early phase of the COVID-19 pandemic, severe disruptions to content and quality of PNC continued to exist, whereas disruptions in availability and use were less commonly reported*” (line 43 - 46).

**Line 344 it states: “Therefore, any further decrease in effective PNC coverage and quality could contribute to increased undetected preventable morbidities among mothers and newborns, and risk losing the ability of achieving future progress in this area.” It is not clear to me why an increase in morbidities would be undetectable. Could you make this clearer. Secondly, what mechanism would cause a risk of losing the ability to achieve future progress once things get back to ‘normal’? Again, a bit more explanation might make the proposed links clearer.**

- The intended message behind the first section of that sentence was that if PNC coverage declines, preventable morbidities would not be detected early or would be detected late (and not that an increase would be undetected). This was clarified in-text: “*Therefore, any further decrease in effective PNC coverage and quality could contribute to increased (preventable) morbidities among mothers and newborns due to insufficient early detection.”* (line 370 - 372).
- We also added this sentence to clarify the link to potential future disruptions in progress: “*Disruptions to PNC seem to be accumulating over the period of the pandemic, which is likely to extend for a long time. This adds a layer of protracted challenges that have to be overcome to achieve any future progress in this area.*” (line 363 - 365)

**Line 388 and accompanying paragraph. Some information seems repeated here (e.g. consequences of reduction in contact with companions mentioned across five/six sentences.) If there is any need to reduce word-count it might be achieved by combining/condensing this section.**

- We thank the reviewer for this comment and we revised this section of the discussion and removed/combined redundant ideas and merged sentences (line 427 - 442).

**Line 471: Here limitations are being discussed. “The nature of the survey and using close-ended questions limited……length of stay questions. It seems like the survey would be something that should be partly under the control of the researchers and could be captured in close-ended questions anyway. Perhaps it would be better just to acknowledge that length of stay questions would be good to incorporate in the future?**

- We have edited this section as follows to reflect the reviewer’s perspective which we agree with: “*The way in which the survey was designed limited the opportunity to have additional useful details, particularly on the new length-of-stay in the hospital after birth, or about the exact context in which the respondents work, such as previous length-of-stay before the pandemic, previous practices related to birth companions and visitors, etc. Future research should include questions that explore these areas in more detail.*” (line 509 - 511)

**I enjoyed reading this paper and I think it makes a good contribution. Thanks for your hard work.**

- Thank you, we appreciate the feedback which significantly improved the quality of the paper.
