# Supplementary material for: “Separated during the first hours”—Postnatal care for women and newborns during the COVID-19 pandemic: A mixed-methods cross-sectional study from a global online survey of maternal and newborn healthcare providers
Source: PLOS Glob Public Health. 2022 Apr 28;2(4):e0000214. doi: 10.1371/journal.pgph.0000214 (PMC10022345; doi:10.1371/journal.pgph.0000214)
Supplement: Attachment — Submitted filename: Point-by-point response_Revision Jan 2022.docx [file pgph.0000214.s005.docx]

**Point-by-point response – 20 January 2022**

**Journal Requirements:**

1. Please update your Competing Interests statement. If you have no competing interests to declare, please state: “The authors have declared that no competing interests exist.”

**Response**: Thank you. the competing interest statement has been updated as suggested.

2. Please review your reference list to ensure that it is complete and correct. If you have cited papers that have been retracted, please include the rationale for doing so in the manuscript text, or remove these references and replace them with relevant current references. Any changes to the reference list should be mentioned in the rebuttal letter that accompanies your revised manuscript. If you need to cite a retracted article, indicate the article’s retracted status in the References list and also include a citation and full reference for the retraction notice.

**Response**: Thank you. We reviewed the reference list one by one and updated the links to certain websites and included DOI when relevant. Please note that no retracted articles were cited, the errors were stemming from broken links or links that were removed or changed by the publisher.

3. While revising your submission, please upload your figure files to the Preflight Analysis and Conversion Engine (PACE) digital diagnostic tool, <https://pacev2.apexcovantage.com/>. PACE helps ensure that figures meet PLOS requirements. To use PACE, you must first register as a user. Registration is free. Then, login and navigate to the UPLOAD tab, where you will find detailed instructions on how to use the tool. If you encounter any issues or have any questions when using PACE, please email PLOS at [figures@plos.org](mailto:figures@plos.org). Please note that Supporting Information files do not need this step.

**Response**: The figure files have been successfully uploaded to the the Preflight Analysis and Conversion Engine (PACE) digital diagnostic tool and updated on the PLOS submission portal.

**Reviewer comments**

**Minor revision:**

**Reviewer #2**: Many thanks for the revised version of the manuscript, which addresses all of the reviewers' comments. I am pleased that you have included absolute numbers and checked the percentages. There is only one minor point to consider left. Table 1 now includes the numbers of data available for each section. Please also update the numbers in the header of the table (n=193 etc). You may further include this information for each section of the table. Many thanks!

**Response**: We thank the reviewer for their comment and thorough revisions. We agree with their suggestion and have added the total numbers of available data at the top of each category in Table 1. We kept the numbers at the header of the table as they are because they represent the distribution of the complete sample by country income group (without missingness by variables).

**Reviewer #3**: It is a study conducted painstakingly. The authors have answered all the queries put forward by the reviewers in a detailed manner. However the study is quite exhaustive and after reading this article, i am confused as to what message are the authors trying to put forth. It would be better if the conclusion is more precise as to throw points which will help in clinical practice.

**Response**: Many thanks for your feedback. We have revised the conclusion and narrowed the focus of the recommendations made. We note that our recommendations are not purely clinical in nature as a result of the collected data which focus more on aspects of the health system (knowledge availability, adherences to guidelines, policies, and emergency preparedness) related to postnatal care provision in times of disruption to functioning health system, such as the COVID-19 pandemic. We hope that the made changes are sufficient to clarify the main points of this work.
